# Uncommon Cardiac Myxoma Arising from the Right Ventricle—Imaging Insights

**DOI:** 10.1016/j.cjco.2025.03.025

**Published:** 2025-04-16

**Authors:** Simon Viscogliosi, Ahmad Hayek, Salim Si-Mohamed, Thomas Varin, Thomas Bochaton

**Affiliations:** a Institut de Cardiologie, Hospices Civils de Lyon, Bron, Lyon, France; b Université de Lyon, INSERM UMR 1060 (CarMeN), Iris, Lyon, France; c Department of Cardiovascular and Thoracic Radiology, Hospices Civils de Lyon, Bron, Lyon, France; d Université de Lyon, CREATIS, UMR 5220, Lyon, France; e Cardiothoracic Surgery Unit, Hospices Civils de Lyon, Bron, Lyon, France

**Cardiac myxomas are the most common type of primary cardiac tumour in adults, but their occurrence in the right ventricle is rare.** **^1^** **This case describes a 64-year-old woman presenting with progressive dyspnea, who was ultimately diagnosed with a large right ventricular (RV) myxoma. Multimodal imaging was crucial in characterizing the mass and differentiating it from malignant tumours. This case highlights the challenges in diagnosing atypical cardiac masses, and it underscores the importance of multimodal imaging and surgical intervention in managing RV myxomas.**

## Clinical Context and Presentation

A 64-year-old woman was admitted to our department for the evaluation of a newly discovered right intraventricular mass. Her significant medical history included a left breast adenocarcinoma, which was treated in 2007 with tumour resection, lymph node dissection, chemotherapy, radiotherapy, and hormone therapy. She has been in remission since then. Her only cardiovascular risk factor was hypertension.

The patient’s symptoms began in early 2024, with progressively worsening dyspnea over the course of 5 months. The dyspnea was isolated and was not associated with general deterioration, anorexia, or weight loss. Her symptoms initially were attributed to infections and were unsuccessfully treated with antibiotics and corticosteroids.

She eventually presented to the emergency department of a regional hospital due to worsening symptoms, including ambient air oxygen desaturation to 86%. A contrast-enhanced thoracic computed tomography (CT) scan revealed a large right intraventricular mass. She was then referred to the cardiology institute of the University Hospital Center in Lyon for further evaluation.

## Clinical Examination and Investigations

The patient was hemodynamically stable upon admission. Her clinical examination was unremarkable, with no signs of heart failure. The electrocardiogram showed a sinus tachycardia at 110 beats per minute, ST-segment depression in the inferior leads, and a prolonged QT interval (QTc: 550 ms).

The laboratory findings revealed hyponatremia, with a sodium level of 131 mmol/L and a borderline low potassium level of 3.5 mmol/L. Her renal function was normal, with an estimated glomerular filtration rate > 90 mL/min per 1.73 m^2^. Inflammatory markers were elevated, with a C-reactive protein (CRP) level of 216.2 nmol/L, and a fibrinogen level of 8.41 g/L. The leukocyte count was within normal limits, at 7.12 × 10^9^/L, and a normocytic anemia was observed, with a hemoglobin level of 115 g/L. Her lactate dehydrogenase levels were normal, at 4466.7 nkat/L. Tumour markers, including carcinoembryonic antigen and cancer antigen 15-3, were within normal reference ranges.

### Etiologic workup

Transthoracic echocardiography revealed a large right intraventricular mass prolapsing into the tricuspid valve, without regurgitation or obstruction, with an extension to the right atrium (Supplemental Fig. S1). The right ventricle was not dilated, and its function remained preserved.

**Figure 1:**
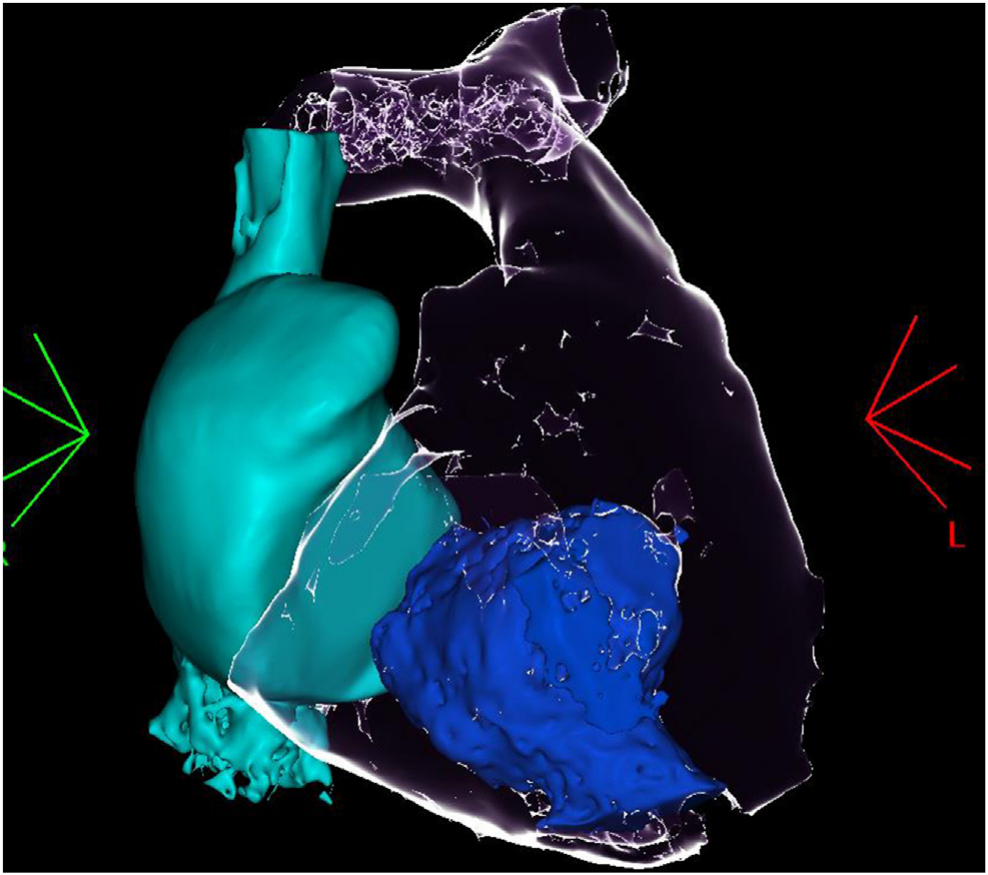
Computed tomography—3-dimensional reconstruction of the right heart chamber. Light blue, right atrium; dark blue, right ventricul mass; transparent, right ventricle.

A contrast-enhanced thoracic CT scan with volumetric reconstruction (Fig. 1; Supplemental Fig. S2) and cardiac magnetic resonance (CMR) imaging (Supplemental Fig. S3) confirmed the presence of an RV mass measuring 30 × 60 mm.

The mass appeared as hyperintense on short inversion time inversion recovery (STIR) sequences, and hypointense on T1-weighted images; it showed heterogeneous enhancement after contrast injection. The mass also infiltrated the RV apex, and extended into the RV outflow tract without involving the infundibulum or pulmonary artery.

The diagnostic workup included a positron emission tomography (PET)-CT scan using fluorodeoxyglucose (FDG; Supplemental Fig. S4), which showed moderate and slightly heterogeneous uptake of the hypodense right intracardiac mass, with a maximum standardized uptake value of 3.2 (< 1.5 times the hepatic background activity). Multiple cardiac biopsies were inconclusive, leaving a differential diagnosis between myxoma and myxoid sarcoma.

The patient underwent cardiac surgery under extracorporeal circulatory assistance (aortic and bi-caval cannulation). The surgical approach was performed via a right longitudinal atriotomy.

The intraoperative examination revealed a supra-centimetric tumour with a base implanted at the RV apex, occupying almost the entire RV cavity and prolapsing through the tricuspid valve. Notably, the mass was enveloping the valvular chordae, and many of them were ruptured. Two samples were taken for pathologic analysis, and the extemporaneous examination confirmed the myxoma.

The tumour was completely removed by eversion of the right ventricle, without the need for ventriculotomy or RV plasty. Due to the severe valvular damage, the decision was made to proceed with a replacement using a biological prosthetic valve.

**Figure 2:**
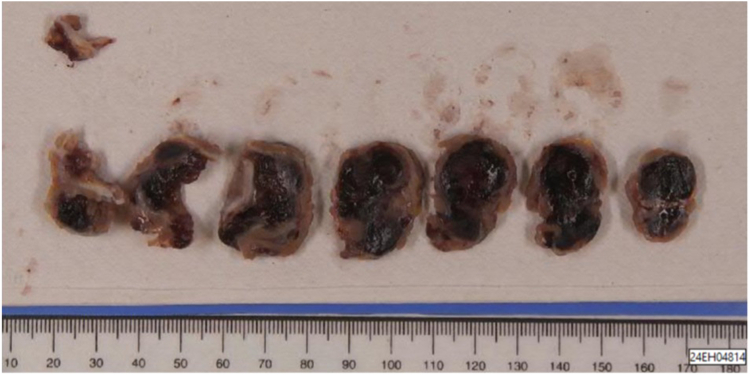
Macroscopic section of the mass.

The macroscopic appearance of the tumour was suggestive of a myxoma. The histologic examination of the entire mass definitively confirmed the diagnosis of myxoma (Fig. 2).

After the surgery, the patient had no residual valvular dysfunction. However, she developed RV failure associated with a slow junctional escape rhythm, prompting the implantation of a leadless pacemaker. The ventricular dysfunction recovered with heart failure treatment.

At the 3-month follow-up evaluation, her dyspnea had improved following cardiac rehabilitation. By the 9-month follow-up evaluation, her symptoms had completely resolved, and echocardiography showed no sign of recurrence.

## Discussion

This case illustrates the challenges of diagnosing and managing cardiac masses, particularly in distinguishing benign from malignant tumours.

The differentiation between benign and malignant cardiac tumours relies on multimodal imaging, primarily echocardiography, CMR, CT, and PET-CT.^2^^,^^3^ Benign tumours are typically well-circumscribed, noninvasive, and mobile on echocardiography, with homogeneous echogenicity. CMR often reveals a well-defined mass with no or minimal contrast enhancement and no signs of tissue infiltration. In particular, myxomas may exhibit heterogeneous enhancement due to hemorrhagic or necrotic areas.

In contrast, malignant cardiac tumours tend to be irregular, infiltrative, and poorly defined. Echocardiography frequently shows an ill-defined, fixed mass with pericardial involvement or chamber obstruction. On CMR imaging, malignant tumours demonstrate heterogeneous signal intensity, extensive late gadolinium enhancement, and necrotic or hemorrhagic components. PET-CT is particularly useful for distinguishing malignancies, as they exhibit high metabolic activity with intense FDG uptake, whereas benign tumours, such as myxomas, generally show low or no FDG uptake.

In our case, multimodal imaging was crucial, but it did not eliminate the diagnostic uncertainty. The RV myxoma exhibited heterogeneous enhancement on CMR imaging, and PET-CT showed moderate FDG uptake, raising the possibility of a malignant process. Given this uncertainty, a definitive diagnosis required surgical excision and histopathologic examination.

The standard treatment for cardiac myxomas is surgical resection due to the risks of obstruction, embolism, arrhythmias, and heart failure. RV myxomas are rare, as the left atrium is the most common location for involvement. The recurrence of sporadic myxomas is rare (< 0.5% per year), but echocardiographic follow-up remains essential.^1^^,^^2^^,^^4^

In our case, the patient did not have a true RV outflow tract obstruction but rather a reduction in RV ejection volume, due to the mass occupying almost the entire ventricular cavity, which likely explained her dyspnea. The patient underwent transthoracic echocardiography at 3 and 9 months postoperatively. No recurrence was observed, which is consistent with reported outcomes in sporadic myxoma cases.

**Novel Teaching Points**

• Multimodal imaging, including echocardiography, CT, CMR, and PET, is crucial for assessing cardiac masses, especially those in atypical locations.
• A multidisciplinary approach, with collaboration among cardiologists, imaging specialists, surgeons, and pathologists, is key to the accurate diagnosis and appropriate management of complex cardiac tumours.
