# Supplementary material for: Uncommon Cardiac Myxoma Arising from the Right Ventricle—Imaging Insights
Source: CJC Open. 2025 Apr 16;7(6):740–2. doi: 10.1016/j.cjco.2025.03.025 (PMC12198621; doi:10.1016/j.cjco.2025.03.025)
Supplement: Supplemental Figures S1-S4 [file mmc1.pdf]

SUPPLEMENTARY MATERIAL

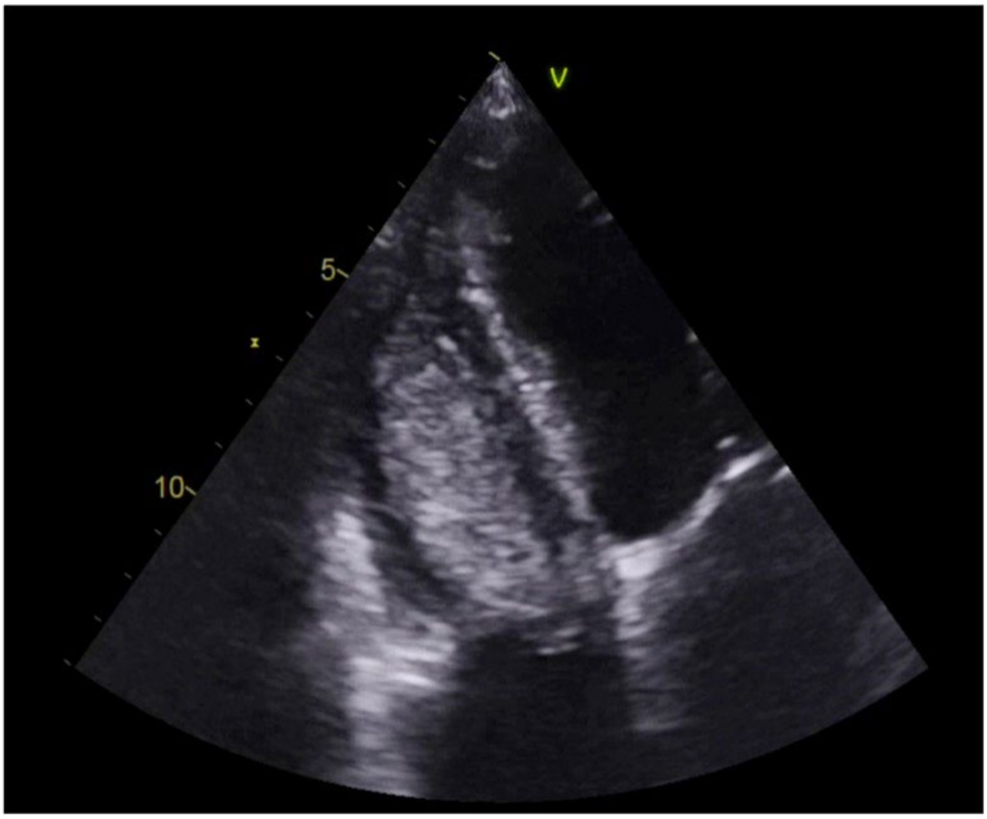

Supplemental Figure S1 : Transthoracic echocardiography.

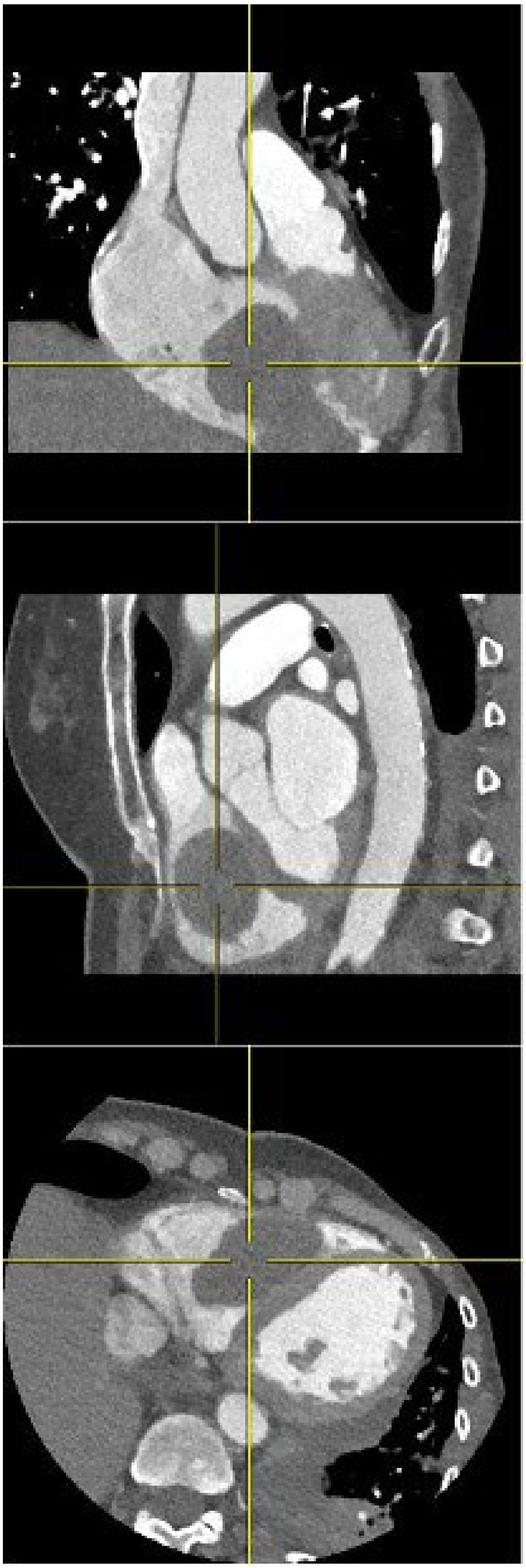

Supplemental Figure S2 : computed tomography scan - MPR

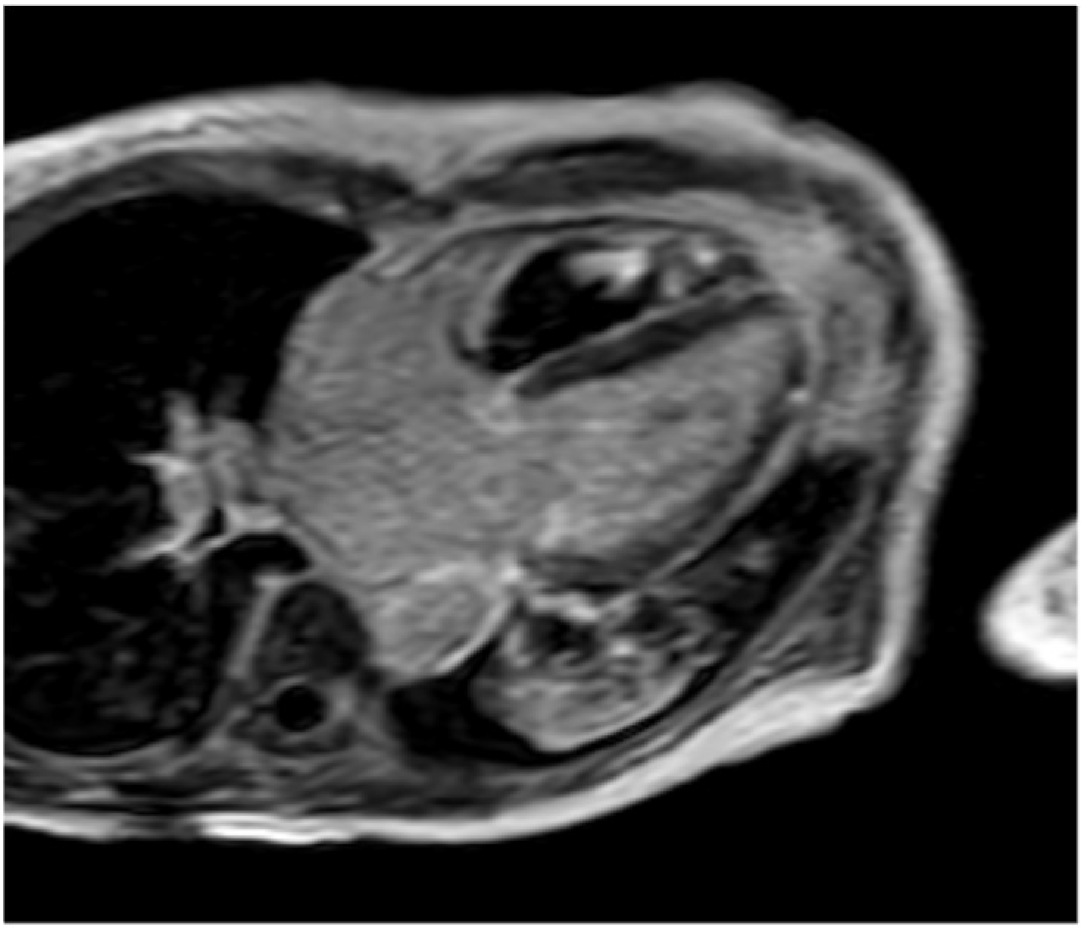

Supplemental Figure S3 : Cardiac magnetic resonance (Late gadolinium enhancement sequence).

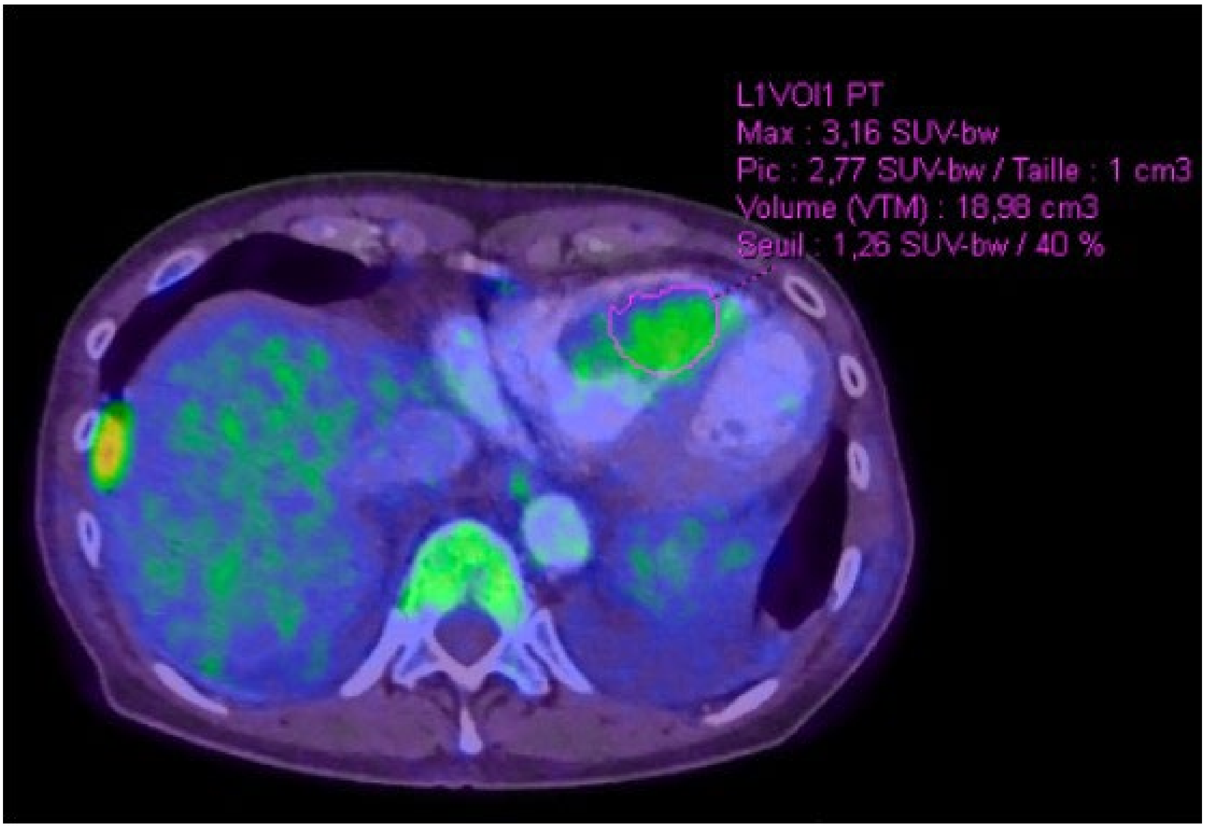

Supplemental Figure S4 : PET-CT scan.
